# Altered Circulating microRNAs in Patients with Diabetic Neuropathy and Corneal Nerve Loss: A Pilot Study

**DOI:** 10.3390/jcm11061632

**Published:** 2022-03-16

**Authors:** Adnan Khan, Jennifer Pasquier, Vimal Ramachandran, Georgios Ponirakis, Ioannis N. Petropoulos, Omar Chidiac, Binitha Thomas, Amal Robay, Amin Jayyousi, Jassim Al Suwaidi, Arash Rafii, Robert A. Menzies, Talal K. Talal, Seyed Hani Najafi-Shoushtari, Charbel Abi Khalil, Rayaz A. Malik

**Affiliations:** 1 Department of Medicine, Weill Cornell Medicine-Qatar, Doha P.O. Box 24144, Qatar; adk2018@qatar-med.cornell.edu (A.K.); gep2011@qatar-med.cornell.edu (G.P.); inp2002@qatar-med.cornell.edu (I.N.P.); 2 Faculty of Health Sciences, Khyber Medical University, Peshawar P.O. Box 25100, Pakistan; 3 Epigenetics Cardiovascular Laboratory, Department of Genetic Medicine, Weill Cornell Medicine-Qatar, Doha P.O. Box 24144, Qatar; pasquiej@gmail.com (J.P.); omc2002@qatar-med.cornell.edu (O.C.); bmt2002@qatar-med.cornell.edu (B.T.); amr2018@qatar-med.cornell.edu (A.R.); jat2021@qatar-med.cornell.edu (A.R.); 4 MicroRNA Core Laboratory, Research Division, Weill Cornell Medicine-Qatar, Doha P.O. Box 24144, Qatar; vir2014@qatar-med.cornell.edu (V.R.); han2005@qatar-med.cornell.edu (S.H.N.-S.); 5 Department of Cell and Developmental Biology, Weill Cornell Medicine, New York, NY 10065, USA; 6 Hamad Medical Corporation, Doha P.O. Box 24144, Qatar; ajayyousi@hamad.qa (A.J.); jalsuwaidi@hamad.qa (J.A.S.); rmenzies@hamad.qa (R.A.M.); ttalal@hamad.qa (T.K.T.); 7 Joan and Sanford I. Weill Department of Medicine, Weill Cornell Medicine, New York, NY 10065, USA

**Keywords:** axonal guidance, corneal nerve, corneal confocal microscopy, diabetic neuropathy, epigenetics, inflammation, microRNAs

## Abstract

An alteration in circulating miRNAs may have important diagnostic and therapeutic relevance in diabetic neuropathy. Patients with type 2 diabetes mellitus (T2DM) underwent an assessment of neuropathic symptoms using Douleur Neuropathique 4 (DN4), the vibration perception threshold (VPT) using a Neurothesiometer, sudomotor function using the Sudoscan, corneal nerve morphology using corneal confocal microscopy (CCM) and circulating miRNAs using high-throughput miRNA expression profiling. Patients with T2DM, with (*n* = 9) and without (*n* = 7) significant corneal nerve loss were comparable in age, gender, diabetes duration, BMI, HbA_1c_, eGFR, blood pressure, and lipid profile. The VPT was significantly higher (*p* < 0.05), and electrochemical skin conductance (*p* < 0.05), corneal nerve fiber density (*p* = 0.001), corneal nerve branch density (*p* = 0.013), and corneal nerve fiber length (*p* < 0.001) were significantly lower in T2DM patients with corneal nerve loss compared to those without corneal nerve loss. Following a q-PCR-based analysis of total plasma microRNAs, we found that miR-92b-3p (*p* = 0.008) was significantly downregulated, while miR-22-3p (*p* = 0.0001) was significantly upregulated in T2DM patients with corneal nerve loss. A network analysis revealed that these miRNAs regulate axonal guidance and neuroinflammation genes. These data support the need for more extensive studies to better understand the role of dysregulated miRNAs’ in diabetic neuropathy.

## 1. Introduction

Epigenetics is the study of heritable phenotype changes in gene activity and function that are not due to alterations in the DNA sequence [1]. Among the three epigenetic mechanisms, non-coding RNAs are known to modulate critical biological processes [2]. Mounting evidence suggests that micro-RNAs (miRNAs) are involved in the pathophysiology of cardiovascular disease, diabetes, and their related complications [3,4] and that circulating miRNAs may also act as biomarkers of disease and therapeutic targets [5].

Diabetic peripheral neuropathy (DPN) affects 30–50% of people with diabetes and can lead to neuropathic pain, foot ulceration, and ultimately, amputation [6]. Its underlying pathogenesis is complex and includes metabolic toxicity, oxidative stress, vascular injury, neurotrophic factor deficiency, and genetic susceptibility [7]. Studies assessing the role of miRNAs have been undertaken predominantly in diabetic mice [8,9], showing an effect on altered axonal growth in the dorsal root ganglion [8] and distal axon [10] and inflammation [11]. Recent studies have reported deregulated miRNAs: mirR-499a [12], mirR-199a-3p [13], mirR-190a-5p [14], miRNA-155 [12], miR-146a [15,16] and miRNA-128a [12,16] in patients with diabetic neuropathy. However, these studies assessed diabetic neuropathy based on relatively crude measures such as clinical history [13], the Toronto Clinical Scoring System and Michigan Neuropathy Screening Instrument [12,15].

Corneal confocal microscopy (CCM) allows for the precise quantification of the severity of diabetic neuropathy [17,18,19]. It has evolved as a powerful tool to diagnose early diabetic neuropathy [20] with a high sensitivity and specificity [21]. We hypothesized that circulating miRNA levels would differ in patients with and without significant corneal nerve loss, assessed using CCM. We further explored potential underlying pathogenetic pathways for diabetic neuropathy.

## 2. Materials and Methods

### 2.1. Participants

Subjects aged 18–85 years old with type 2 diabetes mellitus (T2DM), according to American Diabetes Association criteria [22], were recruited from the diabetes center and podiatry departments at Hamad Medical Corporation in Doha, Qatar. Age, gender, duration of diabetes, height, weight, and BMI were recorded. The systolic (SBP) and diastolic (DBP) blood pressure were assessed in the left arm using a standard zero mercury sphygmomanometer after the subject had been seated for at least 10–15 min, and the average of two readings was obtained. Through venipuncture, 10 mL of blood was collected into vacutainer tubes containing EDTA. The samples were kept at room temperature and transported within 2 h to a central certified laboratory at Hamad General Hospital, HMC, Doha, Qatar. Glycated hemoglobin (HbA_1c_), total cholesterol, LDL, triglycerides, and creatinine were measured by an autoanalyzer (Hitachi 747 Autoanalyzer, Chiyoda, Japan).

Participants with known ophthalmic pathology, auto-immune disease, and peripheral neuropathy (other than diabetic neuropathy), lower limb ischemia, and osteomyelitis were excluded from the study.

### 2.2. Neuropathy Assessment

Vibration perception threshold (VPT) was assessed on the great toe of each foot using a Neurothesiometer (Horwell; Scientific Laboratory Supplies, Wilford, Nottingham, UK). The Sudoscan (Impeto Medical, Paris, France) measured sudomotor function based on sweat chloride concentrations through reverse iontophoresis and chronoamperometry. Patients were asked to place both hands and feet on two nickel electrode plates for 2 min without movement. The ratio between the current generated and the constant DC stimulus (≤4 V) applied to the two sets of electrodes is known as the electrochemical skin conductance (ESC) and expressed in microSiemens (μS) [23]. Corneal confocal microscopy (Heidelberg Retinal Tomograph III Rostock Cornea Module, Heidelberg Engineering GmbH, Heidelberg, Germany) was used to capture central corneal sub-basal plexus nerve images. A drop of local anesthetic (0.4% benoxinate hydrochloride; Chauvin Pharmaceuticals, Chefaro, London, UK) was instilled in each eye, followed by a drop of Viscotears (Carbomer 980, 0.2%, Novartis UK, London, UK) to act as a coupling agent between the cornea and confocal microscope. Four to six images per participant were selected based on the quality of depth, contrast, and focus position. Image analysis was performed using validated, purpose-written software (CCMetrics, M. A. Dabbah, ISBE, University of Manchester, Manchester, UK). Corneal nerve fiber density (CNFD) (no/mm^2^), branch density (CNBD) (no/mm^2^), fiber length (CNFL) (mm/mm^2^) were manually quantified according to our established methodology [24]. CNFL is a validated biomarker for the presence and severity of small nerve fiber damage in diabetic peripheral neuropathy [25]. Patients with T2DM were divided into those with (*n* = 9) (CNFL > 2SD of control (*n* = 20)) and without (*n* = 7) (CNFL < 2 SD of control) corneal nerve loss.

### 2.3. MicroRNA Isolation, Profiling, and Analysis

A volume of 2.5 mL of peripheral blood was withdrawn from each participant and collected in serum tubes followed by centrifugation at 3500 RPM for 5 min to obtain serum. Then, 200 μL of the collected serum was used to isolate microRNAs using the Qiagen miRNeasy serum/plasma kit (Cat. #217184). Further steps were carried out following the manufacturer’s instructions. Total RNAs (including miRNAs) were eluted with 14 μL RNase-free water as per the protocol [26].

Since it is challenging to accurately quantify RNA isolated from the serum, equal volumes of total RNA (4 µL per 20 µL of cDNA synthesis reaction volume) were used to generate cDNA using the Exiqon Universal cDNA Synthesis Kit II (Qiagen miRCURY LNA RT Kit, Cat.# 339340) following manufacturer guidelines [27]. Using equal volumes instead of equal concentrations for reverse transcription of RNA derived from serum/plasma has been previously described [28]. cDNA was diluted fifty-fold and mixed in an equal ratio with 2× Exilent SYBR Green master mix (Qiagen miRCURY LNA SYBR Green PCR Kit (4000), Cat.# 339347).

ROX Reference Dye (ThermoFisher Scientific, Cat# 12223012) was added at a concentration of 4 μL/2 mL. In each of the 384 wells of an Exiqon Serum/Plasma Focus microRNA PCR Panel, 10 μL of the mix was then loaded (V4.M) (Qiagen Human Serum/Plasma Focus, miRCURY LNA miRNA Focus PCR Panel, Cat.# YAHS-106YE-2, Product # 339325). The panels were run on a QuantStudio 12K Flex Real-Time PCR System, and data were processed using GenEx analysis software Version 6 (MultiD Analyses AB, Göteborg, Sweden), following instructions in the software manual.

The quality of the RNA was assessed following amplification using the serum-specific miRNA markers, hsa-miR-23a-3p and hsa-miR-451a. Synthetic spike-ins, UniSp6 and cel-miR-39-3p RNA, provided in the Exiqon Spike-in kit (Qiagen RNA Spike-In Kit, for RT, Cat. # 339390) and mixed with the RNA before cDNA synthesis, were used to monitor the integrity of the cDNA. Proper qPCR amplification was ensured by monitoring the amplification of the UniSp3 spike-in already present on the panels.

Cycle threshold (CT) values above 35 were considered background expressions, and those miRNAs were omitted from the analysis. Samples contaminated with cellular miRNAs due to hemolysis were identified by measuring the difference in Ct values (ΔCt > 7) between hsa-miR-23a-3p and hsa-miR-451a and were left out of the analysis. This method was previously proposed and is the most accurate method to detect hemolysis [29,30]. Data were normalized to the mean of all miRNAs expressed in all samples with a Ct < 35. Normalizing to the global mean rather than specific reference genes was proposed as the optimal method for large PCR-based miRNA screening studies [31].

Run-to-run differences among panels were normalized using UniSp3 inter-plate calibration assays present on the panels. To avoid miRNAs with spurious amplification resulting from primer dimers, a no-template negative control was run on a serum/plasma panel, and a ΔCt of 5 between the sample and negative control was set as a cutoff for each assay. After pre-processing was complete, for statistical analysis, samples were divided into two groups: T2DM without corneal nerve loss and T2DM with corneal nerve loss.

Ingenuity pathway analysis (IPA) was used to predict miRNAs targeted by the upregulated miRNAs and the microRNA target filter in IPA enabled prioritization of experimentally validated and predicted miRNA targets.

### 2.4. Statistical Methods

Statistical analysis was performed using IBM SPSS Statistics software Version 24 on Windows. The Kolmogorov–Smirnov test, visual inspection of a histogram, and the standard Q-Q plot were used to look at the normality of the data. Independent samples t-test or chi-square statistical tests were used to compare baseline characteristics between the study groups. Data are expressed as the mean ± standard deviation. The fold change in the miRNAs between T2DM without and with corneal nerve loss was calculated using an unpaired two-tailed t-test. A confidence interval of 95% (*p*-value ≤ 0.05) was used as a cutoff for significance. Analysis of individual microRNAs was carried out using the GraphPad Prism software program. Outliers were assessed and identified by applying Grubbs’ test (alpha = 0.05%).

## 3. Results

**Figure 1:**
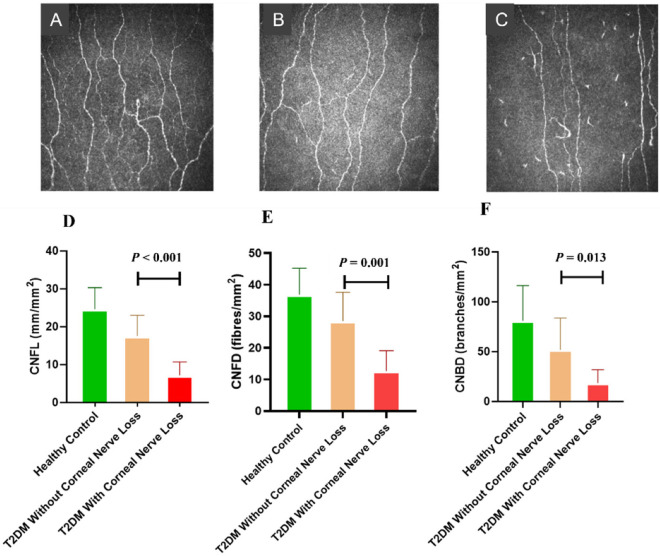
CCM image from a healthy control (**A**), a participant with type 2 diabetes mellitus (T2DM) without corneal nerve loss (**B**), and a participant with T2DM with corneal nerve loss (**C**). There was a significant reduction in CNFL (**D**), CNFD (**E**), and CNBD (**F**) compared to healthy controls (graph represents mean ± SD).

There were no significant differences in age, gender, diabetes duration, BMI, systolic and diastolic blood pressures (BP), HbA_1c_, total cholesterol, LDL-cholesterol, triglycerides, and estimated glomerular filtration rate (eGFR) in T2DM patients with and without corneal nerve loss (Table 1). The DN4 score was comparable, but VPT (*p* < 0.05) was significantly higher; electrochemical skin conductance (ESC) in the feet (*p* < 0.05), corneal nerve fiber length (CNFL) (*p* < 0.001), corneal nerve fiber density (CNFD) (*p* = 0.001) and corneal nerve branch density (CNBD) (*p* = 0.013) were significantly lower in patients with T2DM compared to patients without corneal nerve loss (Table 1, Figure 1).

### 3.1. Serum microRNA Changes Associated with Corneal Nerve Loss

**Figure 2:**
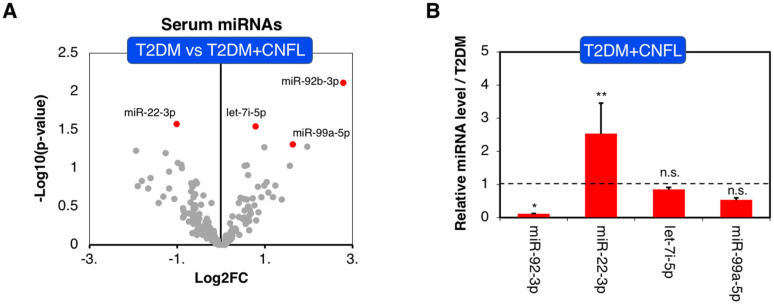
Q-PCR-based serum microRNA expression analysis from T2DM patients with (T2DM) and without significant corneal nerve loss labeled as T2DM and T2DM + CNFL, respectively. (**A**) Volcano plot comparing serum microRNA profiles among T2DM and T2DM + CNFL. The figure illustrates the relationship of FC (log base 2) to the *p*-value (−log base 10). The red dots represent differentially expressed microRNAs with a false discovery rate (FDR) < 0.05 and absolute log2 fold change > 0.5. (**B**) Expression level of the four most significantly altered microRNAs in T2DM + CNFL relative to T2DM (dashed line indicates T2DM level); * *p* < 0.05, ** *p* <0.01. Error bars represent SEM. n.s. represents non-significant (*p* > 0.05).

Based on our initial analysis to determine overall changes in microRNA profiles, we found miR-92b-3p (*p* = 0.008), let-7i-5p (*p* = 0.03), and miR-99a-5p (*p* = 0.05) were significantly downregulated, and miR-22-3p (*p* = 0.03) was significantly upregulated in patients with T2DM compared to patients without corneal nerve loss (Table 2, Figure 2A). However, after a further evaluation of the expression pattern based on mean normalized delta CT values and subsequent testing for potential outliers of each microRNA, we found that only miR-92b-3p and miR-22-3p were significantly altered in relation to corneal nerve loss (Figure 2B and Supplementary Materials S1 and S3), which can partially be explained by the small sample size of the study.

### 3.2. Association of miRNA with Corneal Nerve Parameters

The miR-92b-3p correlated inversely with CNFL (r = −0.69, *p* = 0.013) and CNFD (r = −0.67, *p* = 0.018), and let-7i-5p correlated directly with CNBD (r = 0.556, *p* = 0.031). The association between other miRNAs and clinical parameters is provided in Supplementary Material S2.

### 3.3. Association of microRNA Expression and mRNA via Ingenuity Pathway Analysis

**Figure 3:**
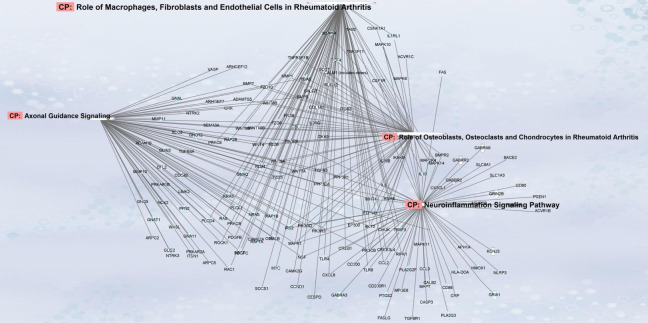
The plot of genes predicted to be affected by the miRNAs differed significantly between patients without and with corneal nerve loss using IPA.

An ingenuity pathway analysis (IPA) was carried out to predict miRNAs targeted by the upregulated miRNAs. The microRNA target filter in IPA provides insights into the biological effects of microRNAs, using experimentally validated interactions from TarBase and miRecords and predicted microRNA–mRNA interactions from TargetScan. The upregulated miRNA identified was projected to target a total of 3480 mRNAs, from which 749 mRNAs were shortlisted by concentrating on pathways of interest for diabetic neuropathy using the microRNA target filter in IPA. Four pathways of interest were identified: the role of macrophages, fibroblasts and endothelial cells in rheumatoid arthritis; axonal guidance; neuroinflammation signaling pathways; and osteoblasts, osteoclasts and chondrocytes in rheumatoid arthritis (Figure 3).

## 4. Discussion

This pilot study has identified the altered expression of specific serum miRNAs and linked this to pathways involved in neuroinflammation and axonal guidance in diabetic patients with corneal nerve loss. We have stratified diabetic patients according to the severity of corneal nerve loss in sub-clinical neuropathy [19,32] and predicted the development of clinical diabetic neuropathy [33,34].

The pathogenesis of diabetic neuropathy is complex with hyperglycemia-driven polyol pathway abnormalities, advanced glycated end products, and dyslipidemia [35]. However, the lack of a single FDA-approved disease-modifying therapy for DPN indicates that other mechanisms warrant further study. In this context, miRNAs are known to modulate altered gene expression and protein synthesis, and their stability in serum allows for a relatively easy detection [36]. Our data suggest that specific miRNAs linked to neuroinflammatory pathways may be relevant in keeping with studies showing elevated cytokine and chemokine production in DPN [37,38,39,40,41]. Increased levels of TNF-α have been associated with a reduction in intraepidermal nerve fiber density and motor and sensory nerve conduction velocities in diabetic animals [38]. Several epidemiological studies have also demonstrated an association between sub-clinical inflammation (CRP, IL6) and diabetic neuropathy using MNSI [42,43]. Reduced nerve conduction velocity has been associated with increased IL6 in patients with type 1 and recently diagnosed type 2 diabetes and early neuropathy [44]. However, in another study, IL18, but not CRP or IL 6, was higher in patients with type 2 diabetes and cardiac autonomic neuropathy [45]. Furthermore, elevated hs-CRP, IL-6, TNF-α, IL-1 receptor antagonist, and soluble intercellular adhesion molecules have been associated with incident diabetic neuropathy [46].

Axonal integrity is key to maintaining nerve function, and altered signaling pathways for axonal guidance have been implicated in diabetic neuropathy [47]. Microarray data from experimental mice with diabetic neuropathy show altered lipid, carbohydrate, and energy metabolism pathways, peroxisome proliferator-activated receptor signaling, and axonal guidance [48]. It is argued that difficulties in demonstrating nerve fiber repair in clinical trials of diabetic neuropathy may result from impaired axonal regeneration [49]. Axonal regeneration can be identified precisely using corneal confocal microscopy; indeed, we show correlations between the expression of specific miRNAs and corneal nerve measures. Furthermore, our IPA shows an association between the altered expression of miRNAs and axonal guidance pathways.

The present study did not identify the upregulation of specific miRNAs found in diabetic patients with DPN based on symptoms and signs [50]. Similarly, miRNA-190a-5p [14] and miR-146a [15] downregulation were associated with increased odds of painful diabetic neuropathy. We believe that our inability to identify comparable alterations in these miRNAs may reflect differences in the populations studied and the relatively crude measures employed to define DPN in these studies which lack precision in identifying underlying axonal pathology, unlike CCM. In animal models, miR-9 mediates neurotransmission [51], and the downregulation of miR-25 leads to an increased expression of advanced end glycation products and worsening diabetic neuropathy [52]. We also recently reported the presence of differentially expressed circulating miRNAs [26] and methylated genes [53] involved in osteoclastic differentiation in patients with diabetic Charcot foot. Although we have identified putative links between the increased expression of specific miRNAs and pathogenetic pathways for diabetic neuropathy, it is important to acknowledge that they are also associated with other diseases. Thus, miR-92b-3p plays a vital role in tumor dynamics and is upregulated in patients with synovial sarcoma and gastric carcinoma.

Furthermore, let-7i-5p was one of seven miRNAs associated with the onset of type 1 diabetes, and miR-99a-5p has a worse prognosis in patients with lung adenocarcinoma. It is also important to acknowledge that most of the circulating miRNAs originate from the liver following cell apoptosis or targeted secretion. Notably, levels of hepatic miR-22-3p are abnormally increased in mouse models of insulin resistance and type 2 diabetes [54]. Therefore, the increase in serum miR-22-3p may also be related to hepatic abnormalities, which are more prevalent in patients with type 2 diabetes and neuropathy.

We acknowledge that this is an exploratory pilot study with a small sample size relative to the large number of miRNAs tested, which increases the risk of spurious correlations. Although our participants were well-matched in clinical and metabolic measures, concomitant treatment could have influenced the expression of miRNAs. The circulating level of miRNAs does not allow us to identify their tissue of origin. Finally, our study population consisted mainly of Arabs; therefore, our results may not be generalized to other ethnic groups.

## 5. Conclusions

In conclusion, we have identified two miRNAs implicated in pathways for neuroinflammation and axonal guidance in diabetic patients with corneal nerve loss. These results provide preliminary data to further investigate the role of miRNAs in relation to diagnostic and therapeutic targets for human diabetic neuropathy. These data also support the need for more extensive longitudinal studies in the future to better understand the role of dysregulated miRNAs’ role as potential biomarkers and therapeutic targets in diabetic neuropathy.

## Figures and Tables

**Table 1:** Clinical, metabolic and neuropathy parameters in patients without and with corneal nerve loss. * *p* < 0.05, ** *p* = 0.001 *** *p* < 0.001.

Characteristics	Without Corneal Nerve Loss	With Corneal Nerve Loss
Number of Participants	7	9
Age (years)	55.43 ± 2.88	57.89 ± 12.61
Gender (male/female)	5/2	5/4
Diabetes duration (years)	11.29 ± 9.34	18.25 ± 9.47
BMI (kg/m^2^)	33.20 ± 3.91	33.25 ± 8.75
SBP (mmHg)	134.14 ± 13.28	131.25 ± 19.84
DBP (mmHg)	77.14 ± 9.75	69.50 ± 3.66
HbA_1c_ (%)	8.47 ± 2.03	8.59 ± 1.86
eGFR (mL/min/1.73 m^2^)	83.66 ± 33.92	70.89 ± 33.86
Total Cholesterol (mmol/L)	4.32 ± 0.92	4.28 ± 1.43
LDL-cholesterol (mmol/L)	2.55 ± 0.82	2.30 ± 1.19
Triglycerides (mmol/L)	1.67 ± 0.98	1.83 ± 0.77
DN4 (score/10)	2.33 ± 2.31	5.88 ± 2.85
VPT (V) *	9.00 ± 6.38	38.50 ± 15.20
ESC feet (µS) *	60.50 ± 29.95	25.38 ± 19.06
CNFL (mm/mm^2^) ***	17.51 ± 5.49	7.11 ± 3.53
CNFD (no./mm^2^) **	28.47 ± 9.15	12.63 ± 6.47
CNBD (no./mm^2^) *	51.81 ± 31.83	18.42 ± 13.50

**Table 2:** Four miRNAs were differentially expressed between T2DM patients with and without corneal nerve loss.

miRNAs	Fold Change	Difference (Log Scale)	*p*-Value
hsa-miR-92b-3p	6.89548	2.78565	0.007694048
hsa-miR-22-3p	−2.01551	−1.01114	0.026614407
hsa-let-7i-5p	1.73163	0.79213	0.028707933
hsa-miR-99a-5p	3.12049	1.64177	0.04869123

## Data Availability

The data presented in this study are available on request from the corresponding author.

## Supplementary Materials

The following supporting information can be downloaded at: https://www.mdpi.com/article/10.3390/jcm11061632/s1. Material S1. Serum microRNA Changes Associated with Corneal Nerve Loss. Material S2. miRNA association with Clinical Parameters. Material S3. Cycle Threshold Values of miRNAs for each Individual Material.
